# Does Beta-lactam Pharmacokinetic Variability in Critically Ill Patients Justify Therapeutic Drug Monitoring? A Systematic Review

**DOI:** 10.1186/2110-5820-2-35

**Published:** 2012-07-28

**Authors:** Fekade Bruck Sime, Michael S Roberts, Sandra L Peake, Jeffrey Lipman, Jason A Roberts

**Affiliations:** 1 School of Pharmacy and Medical Sciences, University of South Australia, Adelaide, Australia; 2 Therapeutics Research Centre, Basil Hetzel Institute for Translational Health Research, The Queen Elizabeth Hospital, Adelaide, Australia; 3 Therapeutics Research Centre, School of Medicine, The University of Queensland, Brisbane, Australia; 4 Department of Intensive Care Medicine, The Queen Elizabeth Hospital, Adelaide, Australia; 5 Burns, Trauma, and Critical Care Research Centre, The University of Queensland, Herston, Brisbane, QLD, Australia; 6 Department of Intensive Care Medicine, Royal Brisbane and Women’s Hospital, Herston, Brisbane, QLD, Australia; 7 Pharmacy Department, Royal Brisbane and Women’s Hospital, Herston, Brisbane, QLD, Australia

**Keywords:** Pharmacokinetics, Pharmacodynamics, Beta-lactam, Antibiotics, Therapeutic drug monitoring, Critically ill

## Abstract

The pharmacokinetics of beta-lactam antibiotics in intensive care patients may be profoundly altered due to the dynamic, unpredictable pathophysiological changes that occur in critical illness. For many drugs, significant increases in the volume of distribution and/or variability in drug clearance are common. When “standard” beta-lactam doses are used, such pharmacokinetic changes can result in subtherapeutic plasma concentrations, treatment failure, and the development of antibiotic resistance. Emerging data support the use of beta-lactam therapeutic drug monitoring (TDM) and individualized dosing to ensure the achievement of pharmacodynamic targets associated with rapid bacterial killing and optimal clinical outcomes. The purpose of this work was to describe the pharmacokinetic variability of beta-lactams in the critically ill and to discuss the potential utility of TDM to optimize antibiotic therapy through a structured literature review of all relevant publications between 1946 and October 2011. Only a few studies have reported the utility of TDM as a tool to improve beta-lactam dosing in critically ill patients. Moreover, there is little agreement between studies on the pharmacodynamic targets required to optimize antibiotic therapy. The impact of TDM on important clinical outcomes also remains to be established. Whereas TDM may be theoretically rational, clinical studies to assess utility in the clinical setting are urgently required.

## Review

### Introduction

Emerging evidence suggests that optimizing antibiotic dosing may be a key intervention to improve outcomes in patients with sepsis and septic shock [1-7]. Nonetheless, dose optimization in this critically ill population remains a significant clinical challenge.

Pathophysiological alterations associated with critical illness can lead to both an increase in the apparent volume of distribution of an antibiotic as well as in clearance; thus potentially leading to subtherapeutic plasma concentrations at the site of infection, treatment failure, and the development of antibiotic resistance [8-11]. Conversely, the development of renal and/or hepatic impairment may be associated with the rapid onset of toxic drug concentrations.

Despite considerable knowledge of the potential issues associated with inadequate antibiotic dosing and the consequences of therapeutic failure, clinicians have little data to guide practice. Although antibiotics are administered frequently in the critically ill [12], to date only a limited number of pharmacokinetic (PK) studies have been undertaken. Instead, the focus has largely been on the avoidance of toxicity from elevated drug concentrations. Accordingly, the available dosing guidelines often are based on PK data obtained from healthy volunteers or noncritically ill patients, with little consideration for the sepsis-induced PK changes that may occur [13]. Moreover, interindividual PK variability in the critically ill, and the consequent unpredictability of drug concentrations, suggests that an empirical fixed dose strategy is unlikely to be successful [14,15].

Although there is a good understanding of the role for therapeutic drug monitoring (TDM) to optimize dosing for drugs with a narrow therapeutic index (e.g., aminoglycosides, glycopeptides), limited data are available regarding TDM of antibiotics with wider therapeutic indices, such as the beta-lactam class of antibiotics [13]. TDM for these antibiotics has been traditionally seen as unnecessary. However, recent data suggest that there is a relationship between beta-lactam antibiotic target exposure and clinical outcomes in the critically ill. In addition, the reported PK variability of these antibiotics suggests that many patients do not achieve their target exposures.

Although published opinion supports the use of TDM to optimize antibiotic therapy for drugs not traditionally subject to TDM (i.e., beta-lactams) [15-17], this role has not been subjected to a structured literature review.

### Purpose

The purpose of this review is to describe the PK variability of beta-lactam antibiotics and to discuss the potential utility of TDM to optimize therapy for critically ill patients.

### Search strategy and results

**Figure 1:**
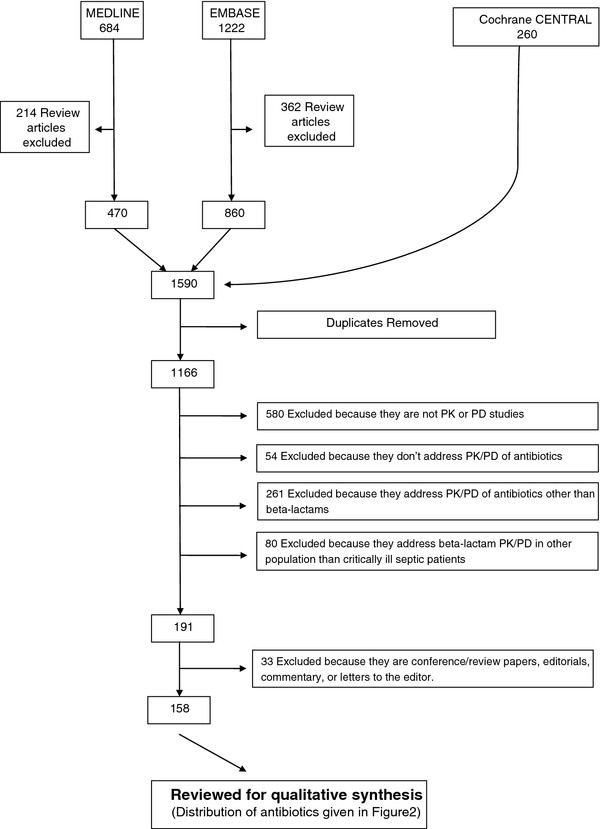
Identification, screening, and selection of articles for the systematic review.

**Figure 2:**
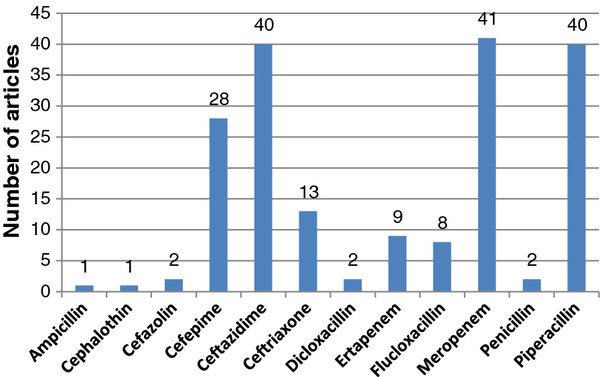
**Number of articles describing pharmacokinetics and pharmacodynamics of selected beta-lactam antibiotics in critically ill patients (relates to Figure**1**).**

Medline (1946 to October 2011), Embase (1947 to October 2011), and the Cochrane Central Registry of Controlled Trials databases were searched for key words to extract data. The search terms were: (1) Pharmacokinetic*, pharmacodynamic*, concentration*, clearance, volume of distribution, target concentration intervention, therapeutic drug monitoring, therapeutic drug management, dosing, dose, kinetics; (2) beta-lactam*, antimicrobial*, antibacterial*, antibiotic*, ampicillin, dicloxacillin, penicillin, flucloxacillin, piperacillin, cephalothin, cefazolin, ceftriaxone, ceftazidime, cefepime, meropenem, ertapenem and; (3) intensive care, critically ill, critical illness, critical care, sepsis, septic shock, h(a)emofiltration, intermittent hemodialysis, extended dialysis, sustained low-efficiency dialysis, slow-flow dialysis. Each search was limited to the English language and human studies. Finally searches (1), (2), and (3) were combined. Studies from the extensive files of the authors also were eligible for inclusion. A total of 158 original research articles describing the PK and pharmacodynamics (PK/PD) of beta-lactam antibiotics in critically ill patients were reviewed for qualitative synthesis (Figure 1). A relatively large number of studies related to the PK/PD of cefepime, ceftazidime, meropenem, and piperacillin in the critically ill. However, there was limited published data for many commonly prescribed antibiotics, which tend to be of narrower spectrum (i.e., ampicillin, cephalothin, cefazolin, ceftriaxone, dicloxacillin, ertapenem, flucloxacillin, penicillin; Figure 2).

### PK/PD variability of beta-lactam antibiotics in the critically ill

Significant, unpredictable, beta-lactam PK variability is well-documented in the critically ill (Table 1). Increase in the volume of distribution (potentially by several-fold) is common [18-27] primarily due to expansion of the extracellular fluid volume (edema). Elimination half-life also can be prolonged due to the increased volume of distribution [21,28-30]. Conversely, clearance may be unchanged [21], decreased [20,22] or even elevated as a result of augmented renal clearance (ARC) in the hyperdynamic phase of sepsis (resulting in sub-therapeutic concentrations of renally cleared antibiotics) [24-26,31]. Enhanced elimination also can be due to a hypoalbuminemia-related reduction in protein binding, particular for highly bound antibiotics, such as flucloxacillin and ceftriaxone [27,28,32].

**Table 1:** Pharmacokinetic parameters of selected beta-lactam antibiotics in critically ill patients without renal dysfunction

**Antibiotic**	**Patients**	**Dose**	**Vd (l/kg)**	**CL (ml/min)**	**T**_**½**_**(h)**	**C**_**max**_**(mg/ml)**	**AUC (mg·h/L)**	**MIC targeted (mg/L)**	**PD target achieved**	**Reference**
Cefepime	12	2 g q12h	0.34*	123	3.2		346	4	77% T > MIC	[18]
	19	2 g q8h	0.36	88.2*	3.37	68	310	32	34% T > 4xMIC	[20]
	13	2 g q12h	0.32*	134	2.5		249			[33]
	7	2 g q12h	0.47*	125	3.42		305	7	80% T > MIC_90_	[19]
								5	100% T > MIC_90_	
	17	2 g q8h	0.37	88.2*	3.37	66.56	324.02			[34]
Ceftazidime	15	2 g q8h	0.81*	151	4.75		277.31			[21]
	12	2 g q8h	0.27*		3.48	124.4	331	4	92% T > MIC	[35]
	17	2 g q8h	0.51	63.7*	6.28	61.65	523.49			[34]
	18	2 g q8h	0.48	112*	5.84	63	522	32	45% T > 4xMIC	[20]
	10	2 g q8h	0.23	112*	1.98					[36]
	49	2 g q8h or 6 g CI/day	0.67*	91.3						[22]
Meropenem	8	2 g q8h or	0.38*	156.7	2.4	110.1	193.8		100% T > MIC	[37]
	7	2 g LD + 3 g CI/day	0.37*	128.3			117.5		100% T > MIC	
	16	1 g q8h	0.43	130.9*	2.05	35	132	8	57% T > 4xMIC	[20]
	10	1 g q8h or 3 g CI/day	0.32*	226.7						[23]
	10	1 g q8h	0.39*	191	2.13	46.6	99.5	0.25-1	100% T > MIC	[38]
Piperacillin	8	12/1.5 g PIP/TAZ CI	0.33	286.7		144				[24]
	8	4/0.5 PIP/TAZ q6h or q8h				266.6				
	27	4 g q6h	0.38	141.4*	2.58	123	469	64	33% T > 4xMIC	[20]
Ceftriaxone	54	2 g qd	0.28*	14.7	9.6			8	16% T > MIC	[25]
	10	2 g qd	0.28*	41.3	6.4	204.9				[30]
Ertapenem	17	1 g CI/day	0.21*	43.2	4.15	90	418	2	25% T > MIC	[39]
	8	1 g qd	0.85*	200.5	5.7	94.1	317.7			[26]
Flucloxacillin	10		0.29*	150.2	2.45					[27]

Vd, volume of distribution; CL, clearance; C_max_, peak serum concentration; AUC, area under the concentration vs. time curve; MIC, minimum inhibitory concentration; CI, continuous infusion; LD, loading dose; PIP, piperacillin; TAZ, tazobactam; *Data were converted considering 70 kg body weight.

Whereas some studies have reported achievement of PD targets in the critically ill with standard beta-lactam dosing strategies used for noncritically ill patients [20,40,41], many others have demonstrated that such empiric antibiotic dosing is insufficient to achieve appropriate PD targets [20,26,27,36,42-48]. These divergent reports reflect the significant interpatient PK variability that occurs in the critically ill. Treatment failure with inappropriate dosing is of particular concern for pathogens with a high minimum inhibitory concentration (MIC) [14,49]. Septic patients also may benefit from higher doses, particularly in the initial 24–48 hours of therapy [38,42,43,50]. Of note, however, even with an increased dose, the attainment of PD targets may still be unreliable, because beta-lactams have predominantly time-dependent kill characteristics, i.e., the time that the unbound (or free) concentration is above the MIC (*f*T_>MIC_) is the major PK/PD index associated with bacterial killing [51-53]. Consequently, the goal of beta-lactam dosing is to optimize the duration of exposure above the MIC during the dosing interval. Increased time above MIC may be achieved by more frequent dosing or by changing the mode of administration from an intermittent bolus injection to either an extended or continuous infusion. The PK of beta-lactam continuous infusions has been most extensively studied for ceftazidime [35,54-59]. However, the clinical superiority of a continuous infusion (versus intermittent injection) for beta-lactams is yet to be established [60].

A further consideration for optimizing antibiotic exposure relates to antibiotic penetration into the interstitial fluid (ISF) of tissues, which is the site of most infections. Data show antibiotic concentrations in ISF that are two- to tenfold lower than plasma concentrations, suggesting that higher plasma concentrations may be required to ensure target concentrations in ISF [11,61,62]. To our knowledge, no studies have adjusted antibiotic doses based on presumed ISF distribution. Problematic to such an approach is that antibiotic concentrations appears to vary between different tissues, suggesting that different plasma target concentrations may be required for the same bacteria depending on the tissue that is the source of infection.

#### PK alterations in acute kidney injury

Acute kidney injury occurs in approximately 5% of critically ill patients and results in significant PK variability for many beta-lactams. Clearance may be extensively reduced, leading to both drug accumulation and toxicity [63-65]. In patients receiving continuous renal replacement therapy (CRRT), extracorporeal clearance is significant, yet variable. Efficient clearance of cefepime [66-68], ceftazidime [69,70], meropenem [71-73], and piperacillin [74,75] has been reported. Ceftriaxone clearance also has been shown to be higher than expected [76,77]. In contrast, CRRT clearance for the highly protein-bound flucloxacillin is minimal [78,79].

It should be noted, however, that it is difficult to compare the reported PK parameters in CRRT studies due to the significant heterogeneity in types of membranes used, operational parameters, and modes of dialysis (Table 2). The consequent unreliable beta-lactam dose prediction for individual patients [80,81] is further compounded by both the limited number of patients studied to date (Table 2) and the fact that different studies cite different PD targets. Finally, the contribution of CRRT to total clearance is variable and dependent on the degree of intrinsic renal function and potential other organ dysfunction [82,83]. As a consequence, there are no definitive dosing guidelines that can be used for all critically ill patients who undergo CRRT. More importantly, some of the current dosing recommendations have been shown to be inadequate, particularly against resistant organisms [69,84], whereas toxicity from unnecessarily high concentrations also has been reported [63-65,82,85].

**Table 2:** Pharmacokinetic parameters of selected beta-lactam antibiotics in critically ill patients undergoing CRRT

**Antibiotic**	**Patients**	**Dose**	**Renal replacement modalities**		**Pharmacokinetic data**										
			**RRT**	**Dialyzer**	**Q**_**B**_**(mL/min)**	**Q**_**UF**_**(mL/min)**	**Q**_**DF**_**(mL/h)**	***C***_**max**_**(mg/L)**	***t***_**H**_**(h)**	***Vd*(l/kg)**	**AUC (mg·h/L)**	***CL***_***tot***_**(mL/min)**	***CL***_**RRT**_**(mL/min)**	**Sc/Sa**	**Ref.**
Cefepime	4	2 g q8h	CVVH or CVVHD	PAN or PS	140-250	16.7-35	500-1000	100.5 l	4.6	0.6		111.5	27.2	0.76	[67]
	5	1-4 g q12h or q24h	CVVH	PAN	150	16		44.6 - 94.9	12.9	0.46	834.7 -1,677.8	35.9	13	0.86	[66]
	7	1-4 g q12h or q24h	CVVHDF	PAN	150	17	857-1020	25.7 -90.8	8.6	0.34	344.9 -1,306.8	46.8	26	0.78	
	8	2 g q12h	CVVH or CVVHDF	AN69	150	25.7*	1610*	43	6.17	0.55	379	72.8*			[84]
Ceftazidime	12	2 g q8h	CVVH	PS	143	47		58.2	4.3	0.52*	344	98.7	32.1	0.69	[70]
	7	3 g q24h	CVVDHF	AN69	150	25	1000		4	0.27*	2514	62	33.6	0.81	[69]
	4	1-2 g q6h	CVVH or CVVHDF	AN69 or PS	130-140	25	500-1000	53.9-112	6.4	0.67		35.5-333.8	5-65.6	0.93	[82]
	12	2 g q12h	CVVH or CVVHDF	AN69	150	25.7*	1610*	78	7.74	0.37	536	36.4*			[84]
Meropenem	8	500 mg q12h	CVVH	AN69	10	26.7		39.5	3.63		105.3	82.94	24.42	0.91	[71]
	5	1 g q12h	CVVH	AN69	150	16.7–33.3			5.16	0.39*	246	4.3	1.96	0.93	[86]
	5	1 g q12h	CVVDHF	AN69	150	16.7 – 25	1000-1500								
	10	1 g q8h	High volume CVVH	AN69	250	66.7-100		56.6	4.3	0.2	166.5	100	58.3	0.93	[72]
	15	0.5-1 g q8h or q12h	CVVHDF	AN 69	90-150	0.17-4.5	600-1500		5.1	0.47*		75	26.7	0.65	[87]
	5	0.5 g q12h	CVVH	PAN	200	25-30		24.5	6.37	0.37	129.5	4.57	1.03	0.63	[88]
	9	0.5 g q8h or q12h	CVVH	AN69	150-170	1.7-2.5		38.9	8.7	0.17*		52	22	1.17	[72]
	9	1 g Stat	CVVH	PS	150	45.8		28.1		0.37*	118	143.7	49.7	0.24	[89]
Piperacillin/ tazobactam	6	4 g q12h/0.5 g q12h	CVVH	PS	100	13.3			7.7/13.9			64.8/40.3			[90]
			CVVHDF				1000		6.7/11.6			84.3/52.2			
							2000		6.1/9.4			91.3/62.5			
	8	2 g/0.25 g or 4 g/0.5 g	CVVHD	AN69	150	1.3-3.3	1500		4.3/5.6	0.31/0.24		47/29.5	22/17	0.87/0.64	[91]
Ceftriaxone	6	2-4 g q24h	CVVH	PA	100-150	20-30			10.8	0.45*			16.6	0.69	[77]
Flucloxacillin	10	4 g q8h	CVVH	PA	169	57		139.1-179.7	4.9	0.69*	568	117.2		0.21	[79]

Q_B_, blood flow rate; Q_UF_, ultrafiltration rate; Q_DF_, dialysate flow rate; RRT, renal replacement therapy; Sc, sieving coefficient; Sa, saturation coefficient; Vd, volume of distribution; CL, clearance; C_max_, peak serum concentration; t_H_, half-life; AUC, area under the concentration vs time curve; CVVH, continuous venovenous hemofiltration; CVVHD, continuous venovenous hemodialysis; CVVHDF, continuous venovenous hemodiafiltration; PAN, polyacrylonitrile; PS, polysulfone; PA, polyamide; *Data were converted considering 70 kg body weight.

### Is there a role for beta-lactam TDM in the critically ill?

#### Do beta-lactams meet the traditional criteria for requiring TDM?

Drugs that are traditionally viewed as appropriate candidates for TDM fulfill one or more of the criteria listed in Table 3. Notably, much of the focus of these criteria is on the prevention of drug toxicity. For many drugs that fulfill the first of these criteria, *a narrow therapeutic index* (e.g., aminoglycosides), TDM is commonly performed. For drugs with a wider therapeutic index, including beta-lactams, TDM has previously been considered to be less clinically relevant because of a lower risk of toxicity. However, TDM may be used, not only to minimize toxicity but also to maximize efficacy [92]. In fact, the primary goal of TDM may be to optimize the clinical response to treatment, with a secondary goal being avoidance of adverse effects.

**Table 3:** **Characteristics of drugs traditionally considered to require TDM**[92,93]

**No.**	**Criteria**
1	Narrow therapeutic range/index
2	Drug toxicity may lead to hospitalization, irreversible organ damage, and even death
3	No clearly defined clinical parameter that allows dose adjustments
4	Correlation exists between serum concentration and efficacy as well as toxicity
5	Unpredictable relationship between dose and clinical outcome
6	Difficult to predict pharmacokinetics (e.g. non-linear pharmacokinetics)

Further rationale for measuring therapeutic beta-lactam concentrations relates to the absence of a *“clearly defined clinical parameter that allows dose adjustments”* (criterion 3). For conventional drugs that are subjected to TDM, such as aminoglycosides, it is difficult to monitor toxicity clinically unless drug levels are monitored, because endpoints for toxicity are poorly defined [92]. Similarly, for beta-lactam antibiotic therapy, there is no established or uniform clinical endpoint that reliably describes resolution of infection and, therefore, adequate dosing. Confirmation of dosing appropriateness using antibiotic concentrations is therefore potentially useful.

The “*relationship between dose and clinical outcome”* for beta-lactams also is unpredictable (criterion 5) due to the variability in clinical response both between and within patients from one dose to the next. A beta-lactam dose that produces a therapeutic effect in one patient may produce toxicity or no clinical response in another patient most probably because of differences in drug distribution into different physiological compartments. Although the PK of beta-lactams in the noncritically ill is generally predictable, the rapidly changing acute pathophysiology and organ dysfunction that occurs in the critically ill means that any assumptions about drug concentrations are unreliable and dose-effect relationships are unpredictable.

Finally, even though “*non-linear PK”* (criterion 6) is not common for beta-lactams, drug accumulation and toxicity can occur due to renal impairment. For example, the accumulation of cefepime in critically ill patients with acute renal failure has been reported to lead to neurotoxicity [63-65,94]. Furthermore, attempts to prevent such toxicity by using standard dose adjustment algorithms [49] have been shown to be unsuccessful. For piperacillin also, similar toxicities have been reported in advanced renal failure at doses conventionally recommended for patients with renal impairment [95,96]. Compressive reviews of neurotoxicity by beta-lactam antibiotics have been published [85,94,97] and emphasize the need for vigilant monitoring.

#### Studies assessing beta-lactam TDM in a clinical environment

Although TDM of beta-lactam antibiotics in the critically ill patient population has previously been called for [27,34,49,91,98], to date, only a few studies describing its utility have been performed [16,45,99-102]. Roberts et al*.* prospectively evaluated TDM in 236 ICU patients and reported that beta-lactam dose adjustment was necessary for 74.2% of patients; 50.4% of the total patients required a dose increment after the first measurement. Their limited outcome findings indicate success rate of 87.3% antibiotic course completion. More recently, the clinical utility of beta-lactam TDM was prospectively examined in a cohort of 50 burn injury patients in a ward environment. For 60% of patients, trough concentrations were less than the target MIC and dose adjustment was required. For patients achieving therapeutic targets, a statistically significant shorter duration of antibiotic therapy was described, thereby demonstrating the therapeutic utility of a TDM program [103].

A prospective study by Aubert et al. [45] assessed serum ceftazidime concentrations in 92 ICU patients. The authors reported that 37% of patients had inadequate ceftazidime concentrations and 27% had excessive concentrations. Ceftazidime dosage was adjusted accordingly to ensure therapeutic concentrations. Connor et al. [104] reported a novel approach for measuring piperacillin-tazobactam drug levels in patients receiving continuous veno-venous hemodialysis (CVVHD) whereby CVVHD effluent was assayed to provide an estimation of plasma drug concentrations for TDM. A piperacillin TDM program in ICU patients using plasma drug concentrations also has been evaluated by Blondiaux et al. [100]. The authors reported that 50% of patients had plasma piperacillin concentrations within the therapeutic target range after continuous infusion of the initial dose and before TDM. Subsequent TDM-guided dose adjustment increased this proportion to 75%.

For meropenem, Taccone et al.[102] recently demonstrated that TDM-guided dose optimization resulted in a successful resolution of sepsis in a patient with extensively drug-resistant *Pseudomonas aeruginosa.* Interestingly, the study demonstrates a rapid emergence of extensively resistant strains most probably due to subinhibitory exposure arising from commonly used dose of 1 g q8h followed by 2 g q8h (MIC was 2 mg/L on day 1, 4 mg/L on day 6, and 8 mg/L a few days later). The investigators performed TDM less frequently (initially on days 2 and 5 of treatment) and were not able to adjust doses early enough to prevent the emergence of resistance and/or therapeutic failure. Their observation, however, presents a novel evidence to suggest frequent TDM: perhaps daily and certainly in the earlier phase of treatment. A case report by Pea et al. [101] also described an intensive TDM process for meropenem and daptomycin.

In summary, the available evidence supporting a beneficial role for beta-lactam TDM in the critically ill is limited. The benefits of TDM may be most evident in patients with severe sepsis as well as in infections with organisms having high MIC. Whether or not TDM-driven dose optimization results in improved clinical outcomes, awaits evidence from a randomized, controlled, clinical trial. It also should be noted that the specified PK/PD target has varied between studies performed to date and to ensure the maximal benefit of TDM an understanding of the appropriate PK/PD targets is essential.

#### Is there a defined PD target for beta-lactam TDM?

For beta-lactams, the best index that describes efficacy is the time the free drug concentration remains above MIC (*f*T_>MIC_) [105]. However, the optimal *f*T_>MIC_ is controversial.

Animal studies suggest that maximal effects can be achieved when *f*T_>MIC_ is less than 100% of the dosing interval, depending on the antibiotic and the organism targeted [51,53,105]. In several studies of cephalosporins, 60–70% *f*T_>MIC_ against *Enterobacteriaceae* and 40–50% *f*T_>MIC_ against *Staphylococcus aureus* have been reported to produce maximal effect [105]. Earlier animal studies also have indicated that *Staphylococci* have maximum killing at 50–60% *f*T_>MIC_, whereas 90–100% *f*T_>MIC_ are required for Gram-negative *Bacilli* and *Streptococci*, presumably due to the absence of a postantibiotic effect [51,53]. For carbapenems, which have relatively high postantibiotic effect, bacteriostatic activity is achieved at 20% *f*T_>MIC_ and bactericidal effects are observed at 40% *f*T_>MIC_[106].

Mouton et al. [107] reported in a dynamic *in vitro* model that maintaining ceftazidime concentration around or slightly above the MIC is not sufficient enough to ensure prolonged efficacy and that targeting *f*T_>4xMIC_ provided sustained and better effect. For meropenem, beneficial outcomes have been observed when the target *f*T_>4-5xMIC_ is maintained [73]. Finally, Tam et al. [108] reported that exposure to 6xMIC is necessary for meropenem to suppress resistance emergence against *P. aeruginosa.*

In patients, TDM using either 100% *f*T_>MIC_[103] or 100% *f*T_>4-5xMIC_[45,99,100,102,103] for several beta-lactams has previously been reported_._ More recently, 54% *f*T_>MIC_ for meropenem has been reported as significant predictor of response in patients with pneumonia [5]. This, and similar studies [7,102,109], suggest that a higher duration of beta-lactam exposure may be required for optimal clinical outcomes than that reported in animal studies. Indeed, this higher exposure may relate to impaired distribution of antibiotic into ISF meaning that a higher plasma exposure is required to achieve an ISF exposure that is equivalent to the animal *in vivo* targets.

Given that many factors can affect the likelihood of a positive outcome in the critically ill, beta-lactam treatment should aim to attain the maximum exposure [79]. Targeting trough concentration (4-5xMIC) may decrease the likelihood of suboptimal plasma concentrations. The higher concentration would enable enhanced distribution of drug into tissues with deranged microcirculation (e.g., septic shock) and improve impaired tissue beta-lactam penetration [11,61,62]. In the absence of well-conducted, prospective, clinical trials addressing the therapeutic benefit of currently recommended PD targets, 100% *f*T_>MIC_ could be considered a prudent PD target for beta-lactams in the critically ill; albeit noncritically ill patients may only require minimal exposures of 40-70% *f*T_>MIC_.

#### Predicting MIC for TDM

The MIC of a target organism(s) is usually not available upon initiation of therapy and may not become available for 24–72 hours after specimens have been sent to a microbiology laboratory. For this reason, epidemiological data of MICs for pathogens can be useful, although there may be considerable variability in the susceptibility of organisms between different institutions within the same geographic location. In cases when the causative organism has been isolated, MIC for the TDM target could be determined by *in vitro* tests, such as the E-test [102]. If these data are not available, an antibiogram for the local institution should be used. Alternatively national guidelines, such as that produced by The French Microbiology Society’s Antibiotics Committee [100], or other databases, such as The European Committee on Antimicrobial Susceptibility Testing (EUCAST), may be highly useful [45,99,101,103].

In cases in which no organisms are isolated, the susceptibility break point of the least susceptible potential pathogen should be used [99,103]. For example, if *P. aeruginosa* and *K. pneumoniae* are the most common causes of pneumonias in an ICU, and piperacillin/tazobactam is the preferred empiric therapy, the TDM that targets the *P. aeruginosa* MIC (16 mg/L *P. aeruginosa* vs. 8 mg/L *K. pneumoniae*) would be appropriate. Later, dose adjustment could be based on the identified pathogen and associated MIC.

#### Beta-lactam assay for use in a TDM program

High performance liquid chromatography (HPLC) is the most common assay technique used in studies of beta-lactam TDM [16,45,99-102]. Verdier et al. [110] developed an HPLC assay method specifically targeting the needs of routine TDM application, thus enabling simultaneous determination of 12 beta-lactams within 22 minutes. Another robust HPLC method described by McWhinney et al. [111] analyzed 12 antibiotics simultaneously within a 7-minute run time. HPLC is, however, a relatively slow technique that requires extensive sample preparation and clean-up processes and, thus, is not suitable for urgent assay needs [112,113]. The relative cost and requirement of specialized instrumentation also is another drawback compared with other techniques, such as immunochemical assays, which use cheaper, portable, and easy-to-use instrumentations [114]. Immunochemical assays have been used for other antibiotics for which routine TDM is well established, such as aminoglycosides and vancomycin [115,116]. However, the development of such techniques for beta-lactams has been a challenge [117,118]. To date, no technique allows simple and rapid determination of unbound beta-lactam plasma concentration, which is ideally required for TDM.

## Conclusions

There is enormous PK variability of beta-lactam antibiotics in critically ill patients. The majority of evidence suggests that empiric approaches to antibiotic dosing may result in subtherapeutic antibiotic concentrations and treatment failure or the emergence of antibiotic resistance. The available studies also strongly support the need for individualized dose optimization in the critically ill, which supports the need for TDM. Despite the theoretical advantages, there remains no consistent use of agreed PK/PD targets. Furthermore, none of the studies have defined the impact of TDM on clinical outcome.

## Abbreviations

ARC, augmented renal clearance; CRRT, continuous renal replacement therapy; CVVHD, continuous veno-venous hemodialysis; fT>MIC, time the free drug concentration is greater than MIC; fT>4xMIC, time the free drug concentration is greater than four times MIC; fT>4-5xMIC, time the free drug concentration is greater than four to five times MIC; ISF, interstitial fluid; MIC, minimum inhibitory concentration; PD, pharmacodynamic; PK, pharmacokinetic; PK/PD, pharmacokinetics and pharmacodynamics; TDM, therapeutic drug monitoring.

## Competing interests

All authors declare that they have no competing interests.

## Authors’ contributions

All authors contributed to drafting of the manuscript and approved the final version.
